# Exposure of Domestic Cats to Three Zoonotic *Bartonella* Species in the United States

**DOI:** 10.3390/pathogens10030354

**Published:** 2021-03-17

**Authors:** Lynn M. Osikowicz, Kalanthe Horiuchi, Irina Goodrich, Edward B. Breitschwerdt, Bruno Chomel, Brad J. Biggerstaff, Michael Kosoy

**Affiliations:** 1 Division of Vector-Borne Diseases, Centers for Disease Control and Prevention, Fort Collins, CO 80521, USA; jqe2@cdc.gov (K.H.); oaq3@cdc.gov (I.G.); bkb5@cdc.gov (B.J.B.); 2 Comparative Medicine Institute, College of Veterinary Medicine, North Carolina State University, Raleigh, NC 27607, USA; ebbreits@ncsu.edu; 3 Department of Population Health and Reproduction, University of California at Davis School of Veterinary Medicine, Davis, CA 95616, USA; bbchomel@ucdavis.edu; 4 KB One Health LLC, Fort Collins, CO 80521, USA; kosoymichael@gmail.com

**Keywords:** domestic cats, cat scratch disease, *Bartonella henselae*, *Bartonella clarridgeiae*, *Bartonella koehlerae*, serosurvey, IFA, vector-borne pathogens

## Abstract

Cat-associated *Bartonella* species, which include *B. henselae*, *B. koehlerae*, and *B. clarridgeiae,* can cause mild to severe illness in humans. In the present study, we evaluated 1362 serum samples obtained from domestic cats across the U.S. for seroreactivity against three species and two strain types of *Bartonella* associated with cats (*B. henselae* type 1, *B. henselae* type 2, *B. koehlerae*, and *B. clarridgeiae*) using an indirect immunofluorescent assay (IFA). Overall, the seroprevalence at the cutoff titer level of ≥1:64 was 23.1%. Seroreactivity was 11.1% and 3.7% at the titer level cutoff of ≥1:128 and at the cutoff of ≥1:256, respectively. The highest observation of seroreactivity occurred in the East South-Central, South Atlantic, West North-Central, and West South-Central regions. The lowest seroreactivity was detected in the East North-Central, Middle Atlantic, Mountain, New England, and Pacific regions. We observed reactivity against all four *Bartonella* spp. antigens in samples from eight out of the nine U.S. geographic regions.

## 1. Introduction

Domestic and wild felines are the natural vertebrate reservoirs for several zoonotic *Bartonella* species, which include *B. henselae*, *B. clarridgeiae*, and *B. koehlerae* [1,2]. The cat flea (*Ctenocephalides felis)* serves as the main vector [1,3]. These bacteria have been detected in cats worldwide, with prevalence often correlated with suitable flea habitat [4,5]. *Bartonella* infected cats are typically asymptomatic; however, there is some evidence that *B. henselae* is associated with febrile illness, endocarditis, myocarditis, and ocular disease [6,7,8]. Furthermore, laboratory findings of basophilia have been documented in cats serologically positive for *B. henselae* [9].

These cat-associated *Bartonella* species infect humans exposed to an infected animal or its ectoparasites, typically through a scratch contaminated with infected flea feces [2]. The clinical symptoms associated with a *B. henselae* infection in humans are collectively known as Cat Scratch Disease (CSD). Symptomatic individuals experience fever, headache, and regional lymphadenopathy [10,11]. More severe pathologies include encephalopathy, endocarditis, retinitis, neurologic complications, osteomyelitis, pulmonary disease, optic neuropathy, splenic and hepatic lesions, and splenomegaly [10,12,13,14,15,16,17,18,19]. *Bartonella clarridgeiae* can cause a typical CSD presentation, including symptoms such as inoculation papules, fever, and lymphadenopathy [20]. While not typically considered a CSD agent, *B. koehlerae* can cause a variety of symptoms, including fever, fatigue, muscle and joint pain, neurological complications, blurred vision, hallucinations, and endocarditis [21,22,23,24]. As *Bartonella* research progresses, more information is known about mammalian hosts, ectoparasite vectors, transmission, and clinical symptoms. As the designation of CSD is limited to only two cat-associated *Bartonella* species, exposure to cats or their fleas, and defined symptoms, the term “bartonellosis” is more appropriate as it encompasses a broad range of disease presentation, routes of transmission, and *Bartonella* species.

In 2016, Nelson et al. [11] analyzed insurance data and reported that from 2005 to 2013 an estimated 12,500 Americans per year under the age of 65 received medical attention for CSD, and approximately 500 of these cases annually resulted in hospitalization. Children under the age of 14 accounted for most of the CSD-diagnosed patients (32%) [11]. The reported CSD cases varied regionally and seasonally, with a higher incidence in southern states, potentially reflecting an increased frequency of flea infestations [11].

Serosurveys of cat populations for the presence and infection prevalence of *Bartonella* species can provide important information for predicting the risk of acquiring bartonellosis in an area. In the U.S., the only large-scale regional *Bartonella* serosurvey found around 28% of cats tested positive [5]. Regional differences revealed a higher prevalence rate in areas with a warmer humid climate compared to areas with a cooler or dry climate, corresponding to suitable flea environments [5]. Since that time, smaller localized studies have been performed, which reported *Bartonella* spp. seroprevalence ranging from 6% to 67% [25,26,27].

These studies have provided valuable information on the *Bartonella* exposure of domestic cats; however, they investigated only two of the three known cat-associated *Bartonella* species, *B. henselae* and *B. clarridgeiae*. Additionally, Jameson et al. [5] estimated the prevalence of antibodies against one serotype of *B. henselae*, which was the only variant known at that time. Currently, two predominant types of *B. henselae*, differentiated by genetic and antigenic characteristics, are associated with human illness: *B. henselae* type 1 (type strain Houston-1) and *B. henselae* type 2 (type strain Marseilles) [4,27,28,29]. Studies from Europe and Asia have reported regional differences in the occurrence of the two genotypes in cat populations [3]. Limited information is available on U.S. regional differences in the occurrence and prevalence of *B. henselae* type 1 and *B. henselae* type 2. Guptill et al. [25] reported most *B. henselae* isolates collected from cats in California were type 2, while in Florida, half of the cat isolates were *B. henselae* type 1 and the other half were *B. henselae* type 2. Little information is available on the exposure of domestic cats in the U.S. to *B. koehlerae,* as compared to other cat-associated *Bartonella* species.

The purpose of our study was to update the information on the regional exposure of U.S. domestic cats to cat-associated *Bartonella* species and to assess the frequency of co-exposures or multi-reactivity among the selected *Bartonella* IFA antigens. Our objectives were to evaluate prevalence in domestic cats from different parts of the U.S. of *Bartonella* antibodies to three species and two strains of *Bartonella* antigen (*B. henselae* type 1, *B. henselae* type 2, *B. clarridgeiae*, and *B. koehlerae*) using an indirect immunofluorescence assay (IFA).

## 2. Results

Overall, 23.1% (314 of 1362, 95% CI: 20.9–25.4%) of cat serum samples were seroreactive against at least one of the four cat-associated *Bartonella* antigens at the cutoff titer of ≥1:64. This number decreased to 11.1% (151 of 1362, 95% CI: 9.5–12.9%) and 3.7% (50 of 1362, 95% CI: 2.8–4.8%) when the antibody cutoff titers of ≥1:128 and ≥1:256 were considered reactive, respectively. The regional seroprevalence at the cutoff titers ≥1:64, ≥1:128, and ≥1:256 for all antigens was higher in East South-Central, South Atlantic, West South-Central, and West North-Central regions and lowest in East North-Central, Middle Atlantic, Mountain, New England, and Pacific regions (Table 1).

When evaluating only the reactive sera at the cutoff titer value of ≥1:64, approximately 31.2% (98 of 314, 95% CI: 26.2–36.7%) were reactive to at least one of the *B. henselae* antigens (type 1 or type 2), 36.9% (116 of 314, 95% CI: 31.6–42.6%) were reactive to the *B. koehlerae* antigen, and 8.9% (28 of 314, 95% CI: 6.1–12.8%) were reactive to the *B. clarridgeiae* antigen. The observed seroreactive samples for each of the four antigens tested can be seen in Table 2.

**Figure 1:**
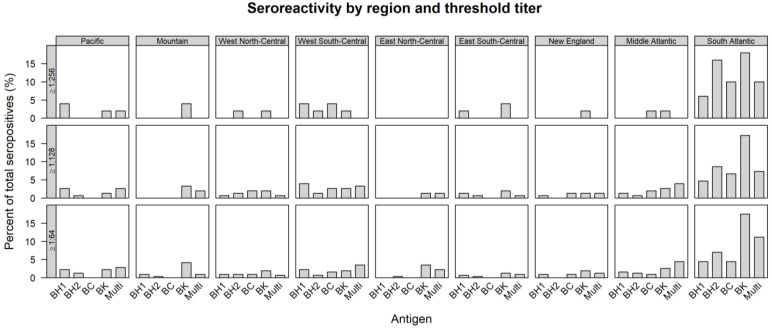
The distribution of seroreactive samples for each antigen at ≥1:64, ≥1:128, and ≥1:256 from each region. *B. henselae* type 1: BH1, *B. henselae* type 2: BH2, *B. clarridgeiae*: BC, *B. koehlerae*: BK, multi-reactive: Multi. The total number of seroreactive cat samples at each titer level are ≥1:256: *n* = 50, ≥1:128: *n* = 151, ≥1:64: *n* = 314.

Sera that were reactive to more than one antigen were classified as multi-reactive. These multi-reactive sera accounted for 6.5% (88 of 1362, 95% CI: 5.2–7.9%), 2.6% (35 of 1362, 95% CI: 1.8–3.6%), and 0.4% (6 of 1362, 95% CI: 0.2–1.0%) of sera at the cutoff titers of ≥1:64, ≥1:128, and ≥1:256, respectively. Only cats from the Pacific (1 of 414) and South Atlantic (5 of 403) regions had multi-reactive samples at a cutoff titer of ≥1:256, and below this titer multi-reactive sera were observed in all regions (Figure 1).

At the cutoff titer of ≥1:64, using results from the Poisson regression model, we estimated that samples had a prevalence ratio of 0.07 (95% CI: 0.03–0.18) of being seroreactive to *B. clarridgeiae* when seroreactive to both *B. henselae* type 1 and *B. henselae* type 2 (where a prevalence ratio <1 indicates less likely to be seroreactive). Considering seroreactivity at titers ≥1:128, there was a slightly lower but similar prevalence ratio of 0.06 (95% CI: 0.03–0.12) of being seroreactive to *B. koehlerae* when already seroreactive to both B. *henselae* type 1 and *B. henselae* type 2 as well as a prevalence ratio of 0.56 (95% CI: 0.0.36–0.88) of being seroreactive to *B. clarridgeiae* when already seroreactive to both *B. henselae* type 1 and *B. koehlerae*. The Poisson regression model was also used to estimate that samples that were *B. henselae* type 1 seroreactive had a prevalence ratio of 0.22 (95% CI: 0.14–0.33) to being reactive to *B. henselae* type 2. Similarly, samples had a prevalence ratio of 0.15 (95% CI: 0.09–0.25) of being seroreactive to *B. koehlerae* infection when already reactive to *B. henselae* type 2 (Table 3).

Excluding samples reactive to more than one IFA antigen, seroreactivity to *B. koehlerae* was found in all regions and *B. koehlerae* was the most reactive antigen, with a total prevalence of 8.5% (116 of 1362, 95% CI: 7.1–10.2%), 3.7% (51 of 1362, 95% CI: 2.8–4.9%), and 1.3% (18 of 1362, 95% CI: 0.8–2.1%) at the cutoff titer values of ≥1:64, ≥1:128, and ≥1:256, respectively (Table 1). Reactivity to *B. henselae* type 1 was observed in all regions except the East North-Central region; *B. henselae* type 2 seroreactivity was observed in all regions except New England; *B. clarridgeiae* reactivity was observed in all regions except East North-Central, East South-Central, Mountain, and Pacific regions. Seroreactivity by region at each titer positivity level is depicted in Figure 1.

In total, there were 1296 (95.1%) samples for which the age of the cat was available and titer could be evaluated. Roughly 3.3% (95% CI: 2.0–5.5%) of kittens/young cats (0–2.9 y), 3.7% (95% CI: 2.4–5.5%) of adult cats (3–10.9 y), and 4.0% (95% CI: 2.3–7.1%) of seniors (11 + y) were reactive for at least one antigen at a titer of ≥1:256. Adult cats accounted for over 40% of the reactive samples at titers of ≥1:64, ≥1:128, and ≥1:256. The titer results by cat age can be seen in Table 4.

From the hurdle model, the type of antigen, age, and region all contributed as predictors to the binomial portion, which modeled the rate ratio (RR) of a sample having positive titer. Compared to *B. henselae* type 1, *B. clarridgeiae* was less likely to result in positive titers, with a RR = 0.75 (95% CI: 0.0.64–0.88), *B. koehlerae* was more likely to result in positive titers, with RR = 1.44 (95% CI: 1.27–1.64), and the *B. henselae* type 2 rate of resulting in positive titers was no different, with RR = 0.92 (95% CI: 0.79–1.08). Age group and region jointly contributed to the probability of a seroreactive titer (*p*-value < 0.001). For the count portion of the model, no predictors statistically significantly contributed, but if a sample crossed the seroreactive threshold, the mean titer was 55.4 (95% CI: 53.3–57.5). 

## 3. Discussion

In this study, the overall seroprevalence against the *Bartonella* spp. antigens of 23.1% at a cutoff titer of ≥1:64 was slightly lower than the 28% overall seroprevalence in U.S. cats reported by Jameson et al. [5]. That study only evaluated titer values of ≥1:64 against *B. henselae* type 1. In our study, if we only evaluate *B. henselae* type 1 reactive and multi-reactive samples at a titer of ≥1:64, the seroprevalence is 7.5% (102 of 1362, 95% CI: 6.2–9.0%).

In our study, *B. koehlerae* was the most reactive antigen at all titer levels, and seroreactivity to it was observed in all regions. The two *B. henselae* antigens were the next most reactive antigens, followed by *B. clarridgeiae*. In dogs, Lashnits et al. [30] reported *B. koehlerae* seroreactivity from all U.S. regions and a similar seroprevalence between *B. henselae* (2.13%) and *B. koehlerae* (2.39%) [30]. Around the world, *B. henselae* antibodies have been detected in cats [3,4]. However, the *B. koehlerae* antigen has been included in few feline serological investigations. In Spain, 41.6% of veterinary personnel were seroreactive against the *B. koehlerae* antigen, while 37% were reactive against *B. henselae*, suggesting *B. koehlerae* is prevalent in Spanish cat or dog populations [31]. A study in Israel, reported *B. clarridgeiae* and *B. koehlerae* to be more prevalent than *B. henselae* in stray cats, using molecular techniques [32]. Future serological studies should include the *B. koehlerae* antigen in order to better understand seroprevalence in cat populations.

**Figure 2:**
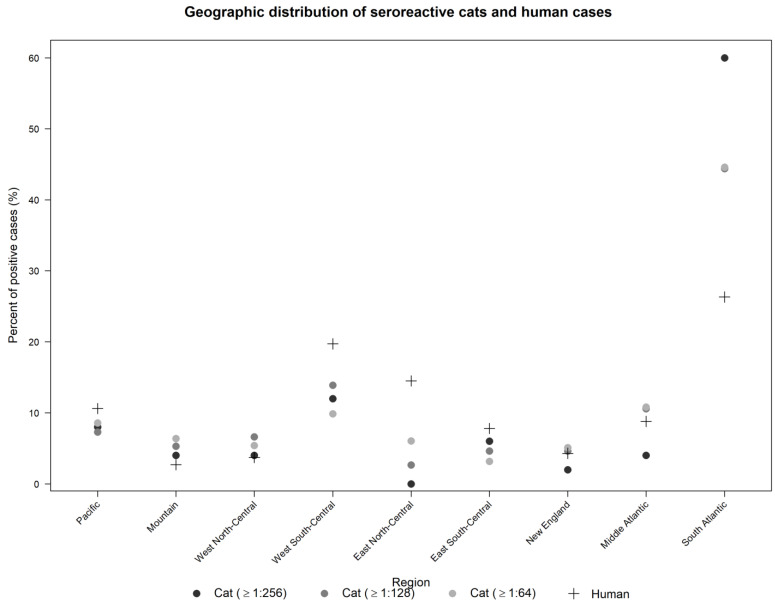
The percent of seroreactive cat samples at the endpoint titers of ≥1:64 (light grey), ≥1:128 (dark grey), and ≥1:256 (black), and the percent of human cases (plus sign) reported by Nelson et al. [11] from each U.S. region. The total number of seroreactive cat samples at each titer level are ≥1:256: *n* = 50, ≥1:128: *n*= 151, ≥1:64: *n* = 314.

Overall, the observed regional variation in seroprevalence, with a higher prevalence at the ≥1:64 titers in the southern regions (East South-Central: 34.5%, South Atlantic: 34.7%, West South-Central: 35.6%) and the West North-Central region (41.5%) than in western regions (Mountain: 20.0%, Pacific: 6.5%), the East North-Central region (20.0%), and northeastern regions (Middle Atlantic: 29.1%, New England: 21.1%). These results are similar to those of Jameson et al. [5], who suggested that seroprevalence in cats is related to areas that support favorable habitat for flea populations, as *C. felis* is the main vector for transmission of these *Bartonella* species among cats and prefers a humid environment [4,33,34]. Similar to the human case reports by Nelson et al. [11], a greater proportion of the seroreactive cat samples in our study occurred in the southern U.S. (Figure 2). This parallel suggests that cat seroprevalence is geographically associated with human cases. However, more research should be performed to investigate this observation. A recent serological survey of U.S. dogs from 2008–2014 evaluated IFA results from three *Bartonella* spp. antigens (*B. henselae*, *B. koehlerae*, *B*. *vinsonii* subsp. *berkhoffii*) [30]. In that study, only 3.26% of 15,451 diagnostic sera were reactive to any one of the three *Bartonella* spp. antigens [30]. The authors did not find any regional differences in seroreactivity, but rather that dogs were broadly exposed to each of the *Bartonella* antigens tested [30]. Further analysis of these data for the state of North Carolina indicated that multiple factors, including the owner’s socioeconomic status, land use, and climate, are associated with seroreactivity in dogs [35]. Future efforts should be made to investigate a larger number of cat samples, particularly in states and regions with low representation.

Although IFA is a common *Bartonella* serological assay, interpreting titer results can be complicated [3,30,36]. Seroreactivity from a serosurvey is typically indicative of a past exposure, and experimental studies have reported seroreactive cats up to 190 days post-infection [4,37]. Some authors have noted that higher antibody titers are associated with bacteremic cats, and lower titers may indicate either slight bacteremia or a past infection [10]. Using current technology, we cannot distinguish whether a cat was co-infected or serially infected with multiple *Bartonella* species, or if their serum is just cross-reactive. A cat can be exposed to multiple *Bartonella* species in its lifetime, and *Bartonella* co-infected cats have been documented [38]. In addition, *B. henselae* and *Bartonella quintana* antigens may cross-react when testing human sera [39], so it is possible that some cats are cross-reactive for multiple *Bartonella* species.

We observed multi-reactive cats ranging from 0.4% at titers ≥1:256 to 6.5% at titers ≥1:64 and found some associations between antigens of multi-reactive samples. For example, a sample reactive for *B. henselae* type 1 was less likely to also be multi-reactive for *B. henselae* type 2, and if a sample was reactive for *B. henselae* type 2 then it was less likely to also be multi-reactive for *B. koehlerae*. A study analyzing the specificity of *Bartonella* antigens in dogs found that experimentally infected animals only reacted to the specific *Bartonella* species they had been infected with and did not cross-react with other *Bartonella* antigens [40]. The authors concluded that multi-reactive samples likely resulted from exposure to multiple *Bartonella* species rather than cross-reactivity [40]. Furthermore, the fact that seroreactivity to three *Bartonella* spp. antigens was less than 3% in 15,451 diagnostic sera submitted by veterinarians suspecting infection with a vector-borne pathogen further supports IFA specificity when testing dogs [30]. Regrettably, *Bartonella* IFA sensitivity is poor when testing dogs with PCR-confirmed infections [41,42]. Thus, epidemiological studies using dog sera should have excellent specificity, but will underestimate exposure among dogs regionally. Multi-reactive samples in our study could represent exposure to multiple *Bartonella* species, cross-reactivity, or differences in criteria for the endpoint titer evaluation.

Several limitations exist in our study. The sample set is not fully representative of the U.S. cat population due to unknown criteria for the selection, cat health data, travel history, and discrepancies in availability of the samples by a region. Specifically, selection bias may exist towards cats that are well cared for because an owner would have to be willing to pay for testing services. Additionally, if the animals are well cared for, they may also be using flea control products. Flea control practices for house cats have likely become more popular since the early 1990s due to increased client education and the development of new flea control products. Since 1997, several new active ingredients and combination ingredients have been registered for flea control and are available for convenient use as oral and topical treatments for companion animals [33]. The use of flea control products has been experimentally shown to prevent *Bartonella* infections in cats [43,44,45]. Clearly, based upon regional and national seroprevalences reported in cats, and the emerging importance of cat-associated *Bartonella* spp. as a cause of human illnesses, there should be increased public health and veterinary emphasis on flea control measures.

Currently, human *Bartonella* diagnostic techniques include IFA testing on acute and convalescent sera, using *B. henselae* and *B. quintana* antigens. Dalton et al. [46], when evaluating the commonly used human diagnostic assay at ≥1:64 titer in patients with suspected CSD, reported a sensitivity of 95% when using a strict clinical definition and 82% for those patients who met a broad case definition. Using an assay that included additional antigens, we show that cats across the U.S. are exposed to all four *Bartonella* antigens at an overall seroprevalence at the ≥1:64 titer of 23.1%. Although the overall seroprevalence is low, we observed exposure to *B. clarridgeiae* and *B. koehlerae,* but not to *B. henselae*, in half of the seroreactive cats across all titer levels examined. Consequently, people may be exposed to *Bartonella* species that may not be detected by traditional *B. henselae* and *B. quintana* IFA assays.

Our results demonstrate differences in seroreactivity of cats to *Bartonella* species; however, more research is needed to investigate this observation and potential association. Possible sample biases in this study have been identified, and future efforts should employ formal, statistical sampling methods to ameliorate these concerns. Furthermore, efforts to increase sample number across regions and to consider the animal’s health when characterizing historical *Bartonella* species exposures would strengthen conclusions. Nevertheless, from this observational study, we observed similarities between cat-associated *Bartonella* species seroreactivity and the overall regional proportion of human CSD cases. We were also able to document cat seroreactivity in the U.S. to *B. koehlerae*, a *Bartonella* spp. that has been associated with human illness but not classical CSD.

## 4. Materials and Methods

### 4.1. Sample Collection

We tested archived cat serum samples, originally submitted for suspected illness or blood donor screenings to three veterinary diagnostic laboratories, located at Colorado State University, the University of California at Davis, and North Carolina State University between 2008 and 2017. In total, 1362 cat serum samples from 38 states and Washington, D.C. were analyzed. The 38 states and Washington, D.C. were grouped to represent the nine geographic regions of the continental U.S., following the scheme used by Nelson et al. [11] (Table 5). Selected samples, stored at −80 °C, were sent to the U.S. Centers for Disease Control and Prevention (CDC) in Fort Collins, Colorado, for serologic testing.

### 4.2. Serology

The cat sera were tested by an IFA. The antigen production, slide preparation, and IFA procedure were performed with slight modifications, according to a previously published protocol [47]. The modifications included production of antigens for each of the selected *Bartonella* species of interest (*B. henselae* type 1, B5344/ATCC 49882, passage 2, isolated from human; *B. henselae* type 2, B44871, passage 2, isolated from cat; *B. clarridgeiae* B30992/ATCC 51734, passage 3, isolated from cat; and *B. koehlerae* subsp. *koehlerae*, B8966/ CCUG 50773, passage 2, isolated from cat). Additionally, the slides were prepared using 48-well microscope slides (Tekdon Inc., Myakka City, FL, USA) and stored at −20 °C for future use. The Vero E6 cell line used to produce the antigen was obtained from the Centers for Disease Control and Prevention, Ft. Collins, Colorado. Positive and negative controls were included with each run. The positive controls against each of the four cat-associated *Bartonella* antigens were custom mouse antibodies created by ProSci Incorporated (Poway, CA, USA). We used anti-cat FITC-labeled IgG (H + L) conjugate (Sera Care, Milford, MA, USA) for samples and anti-mouse FITC-labeled IgG (H + L) conjugate (Sera Care, Milford, CT, USA) for the controls.

Each well was scored for fluorescent reactivity, and any reactive sample at the screening dilution of 1:32 was further diluted twofold until the sample was no longer reactive. The last dilution at which florescence was observed for a specific antigen was recorded as the final cutoff titer. Seroreactive results were evaluated separately by two investigators. For statistical analyses, reactive antibody cutoff titers of ≥1:64, ≥1:128, and ≥1:256 were each evaluated.

### 4.3. Statistical Analysis

To evaluate associations of cat age, region of collection, and antigen type with antibody titers, data were analyzed using a hurdle regression model. The hurdle model has two components, one to model the probability of a sample having a positive titer using logistic regression with a log link, and the other to model the value of the titer among those for which it is positive using a zero-truncated Poisson regression model. Bootstrapping was used to compute 95% confidence intervals (CIs) for model parameters. A Poisson regression model was used to assess cross-reactivity of the four antigens at each of the three cutoff levels. Prevalence ratios (PRs) and 95% CIs were calculated to compare antigen reactivity. Statistical analyses were conducted using the “countreg”, “multcomp”, and “boot” packages in R version 3.6.1 (R Foundation for Statistical Computing, Vienna, Austria).

## Figures and Tables

**Table 1:** Number of serum samples by region reactive against each of the four antigen (*B. henselae* type 1: *B.h*. 1, *B. henselae* type 2: *B.h*. 2, *B. clarridgeiae*: *B.c.*, *B. koehlerae*: *B.k*.) and multi-reactive serum samples (Multi) at titers ≥1:64, ≥1:128, and ≥1:256.

REGION	TOTAL TESTED	≥1:64					≥1:128					≥1:256				
		*B.h.* 1	*B.h.* 2	*B.c.*	*B.k.*	Multi	*B.h.* 1	*B.h.* 2	*B.c.*	*B.k.*	Multi	*B.h.* 1	*B.h.* 2	*B.c.*	*B.k.*	Multi
		*n* (%)	*n* (%)	*n* (%)	*n* (%)	*n* (%)	*n* (%)	*n* (%)	*n* (%)	*n* (%)	*n* (%)	*n* (%)	*n* (%)	*n* (%)	*n* (%)	*n* (%)
Pacific	414	7 (1.7)	4 (1.0)	0 (0.0)	7 (1.7)	9 (2.2)	4 (1.0)	1 (0.2)	0 (0.0)	2 (0.5)	4 (1.0)	2 (0.5)	0 (0.0)	0 (0.0)	1 (0.2)	1 (0.2)
Mountain	100	3 (3.0)	1 (1.0)	0 (0.0)	13 (13.0)	3 (3.0)	0 (0.0)	0 (0.0)	0 (0.0)	5 (5.0)	3 (3.0)	0 (0.0)	0 (0.0)	0 (0.0)	2 (2.0)	0 (0.0)
West North-Central	41	3 (7.3)	3 (7.3)	3 (7.3)	6 (14.6)	2 (4.9)	1 (2.4)	2 (4.9)	3 (7.3)	3 (7.3)	1 (2.4)	0 (0.0)	1 (2.4)	0 (0.0)	1 (2.4)	0 (0.0)
West South-Central	87	7 (8.0)	2 (2.3)	5 (5.7)	6 (6.9)	11 (12.6)	6 (6.9)	2 (2.3)	4 (4.6)	4 (4.6)	5 (5.7)	2 (2.3)	1 (1.1)	2 (2.3)	1 (1.1)	0 (0.0)
East North-Central	95	0 (0.0)	1 (1.1)	0 (0.0)	11 (11.6)	7 (7.4)	0 (0.0)	0 (0.0)	0 (0.0)	2 (2.1)	2 (2.1)	0 (0.0)	0 (0.0)	0 (0.0)	0 (0.0)	0 (0.0)
East South-Central	29	2 (6.9)	1 (3.4)	0 (0.0)	4 (13.4)	3 (10.3)	2 (6.9)	1 (3.4)	0 (0.0)	3 (10.3)	1 (3.4)	1 (3.4)	0 (0.0)	0 (0.0)	2 (6.9)	0 (0.0)
New England	76	3 (3.9)	0 (0.0)	3 (3.9)	6 (7.9)	4 (5.3)	1 (1.3)	0 (0.0)	2 (2.6)	2 (2.6)	2 (2.6)	0 (0.0)	0 (0.0)	0 (0.0)	1 (1.3)	0 (0.0)
Middle Atlantic	117	5 (4.3)	4 (3.4)	3 (2.6)	8 (6.8)	14 (12.0)	2 (1.7)	1 (0.9)	3 (2.6)	4 (3.4)	6 (5.1)	0 (0.0)	0 (0.0)	1 (0.9)	1 (0.9)	0 (0.0)
South Atlantic	403	14 (3.5)	22 (5.5)	14 (3.5)	55 (13.6)	35 (8.7)	7 (1.7)	13 (3.2)	10 (2.5)	26 (6.5)	11 (2.7)	3 (0.7)	8 (2.0)	5 (1.2)	9 (2.2)	5 (1.2)
Total	1362	44 (3.2)	38 (2.8)	28 (2.1)	116 (8.5)	88 (6.5)	23 (1.7)	20 (1.5)	22 (1.6)	51 (3.7)	35 (2.6)	8 (0.6)	10 (0.7)	8 (0.6)	18 (1.3)	6 (0.4)

**Table 2:** Cat serum samples that were seroreactive and multi-reactive (*n*) for each of four test antigens at titers of ≥1:64, ≥1:128, and ≥1:256 over the total number of seroreactive samples. *B. henselae* type 1: *B.h*. 1, *B. henselae* type 2: *B.h*. 2, *B. clarridgeiae*: *B.c*., *B. koehlerae*: *B.k.*

Antigen	≥1:64		≥1:128		≥1:256	
	*n*	%	*n*	%	*n*	%
*B.h.* 1	44	14.0	23	15.2	8	16.0
*B.h.* 2	38	12.1	20	13.2	10	20.0
*B.c.*	28	8.9	22	14.6	8	16.0
*B.k.*	116	36.9	51	33.8	18	36.0
*B.h.* 1, *B.h.* 2	16	5.1	14	9.3	2	4.0
*B.h.* 1, *B.c.*	4	1.3	1	0.7	0	0
*B.h.* 1, *B.k.*	12	3.8	3	2.0	0	0
*B.h.* 2, *B.k.*	15	4.8	6	4.0	2	4.0
*B.h.* 2, *B.c.*	5	1.6	3	2.0	1	2.0
*B.c.*, *B.k.*	7	2.2	2	1.3	1	2.0
*B.h.* 1, *B.h.* 2, *B.c.*	2	0.6	0	0	0	0
*B.h.* 2, *B.c.*, *B.k.*	3	1.0	1	0.7	0	0
*B.h.* 1, *B.c., B.k.*	7	2.2	2	1.3	0	0
*B.h.* 1, *B.h.* 2, *B.k.*	15	4.8	1	0.7	0	0
*B.h.* 1, *B.h.* 2, *B.c., B.k.*	2	0.6	2	1.3	0	0
Total	314		151		50	

**Table 3:** Prevalence ratios (95% CIs) for statistically significant multiple *Bartonella* antigen reactivity. A prevalence ratio <1 indicates lower prevalence when already infected with other antigens. *B. henselae* type 1: *B.h*. 1, *B. henselae* type 2: *B.h*. 2, *B. clarridgeiae*: *B.c*., *B. koehlerae*: *B.k.*

Antigen Reactivity	≥1:64	≥1:128	≥1:256
*B.c.* when infected with *B.h.* 1 and *B.h.* 2	0.07 (0.03–0.18)		
*B.k.* when infected with *B.h.* 1 and *B.h.* 2		0.06 (0.03–0.12)	
*B.c.* when infected with *B.h.* 1 and *B.k*		0.56 (0.36–0.88)	
*B.c.* when infected with *B.h.* 2	0.11 (0.07–0.16)	0.09 (0.04–0.25)	
*B.k.* when infected with *B.h.* 1	0.31 (0.24–0.40)	0.13 (0.11–0.17)	
*B.k.* when infected with *B.h.* 2	0.34 (0.26–0.44)	0.28 (0.23–0.34)	0.15 (0.09–0.25)
*B.h.* 2 when infected with *B.h.* 1	0.37 (0.33–0.42)	0.56 (0.44–0.70)	0.22 (0.14–0.33)
*B.c.* when infected with *B.h.* 1	0.11 (0.07–0.31)	0.01 (0.01–0.23)	
*B.c.* when infected with *B.k.*	0.07 (0.05–0.09)	0.04 (0.03–0.05)	

**Table 4:** Bartonella antibody titer levels detected in cats at each life stage. % life stage is the percent of samples reactive from the total number of samples tested for each life state. % total reactive is the percent of samples reactive from the total number of reactive samples at each titer level. There were 66 samples that did not have age information; the % total reactive denominator does not include these.

Life Stage	Approx. Age	Tested	≥1:64			≥1:128			≥1:256		
			*n*	% Life Stage	% Total Reactive	*n*	% Life Stage	% Total Reactive	*n*	% Life Stage	% Total Reactive
Young Cat	0–2.9 y	448	86	19.2	28.6	49	10.9	34.3	15	3.3	31.9
Adult	3–10.9 y	575	142	24.7	47.2	59	10.3	41.3	21	3.7	44.7
Senior	≥11 y	273	73	26.7	24.3	35	12.8	24.5	11	4.0	23.4

**Table 5:** Number of cat serum samples tested by state in each region.

Pacific		Mountain		West North-Central		West South-Central		East North-Central		East South-Central		New England		Middle Atlantic		South Atlantic	
State	*n*	State	*n*	State	*n*	State	*n*	State	*n*	State	*n*	State	*n*	State	*n*	State	*n*
CA	407	AZ	2	IA	7	AR	16	IL	28	AL	6	CT	9	NJ	2	DC	3
WA	7	CO	88	KS	14	LA	5	IN	7	KY	2	MA	64	NY	97	DE	1
		NM	6	MN	2	OK	5	MI	22	MS	3	NH	3	PA	18	FL	47
		WY	4	MO	17	TX	61	OH	31	TN	18					GA	7
				NE	1			WI	7							MD	21
																NC	264
																SC	14
																VA	45
																WV	1
Total	414	Total	100	Total	41	Total	87	Total	95	Total	29	Total	76	Total	117	Total	403
